# *tert*-Butyl 3-carbamoyl-4-methoxy­imino-3-methyl­piperidine-1-carboxyl­ate

**DOI:** 10.1107/S1600536809000634

**Published:** 2009-01-10

**Authors:** Yun Chai, Zhi-Long Wan, Hui-Yuan Guo, Ming-Liang Liu

**Affiliations:** a Institute of Medicinal Biotechnology, Chinese Academy of Medical Sciences and Peking Union Medical College, Beijing 100050, People’s Republic of China

## Abstract

In the title compound, C_13_H_23_N_3_O_4_, the piperidine ring adopts a chair conformation. An intra­molecular N—H⋯O hydrogen bond is observed between the carbamoyl and carboxyl­ate groups. In the crystal structure, mol­ecules form inversion dimers linked by pairs of N—H⋯O hydrogen bonds.

## Related literature

For the synthesis and properties of quinolone derivatives, see: Anderson & Osheroff (2001 ▶); Ball *et al.* (1998 ▶); Choi *et al.* (2004 ▶); Ray *et al.* (2005 ▶); Wang, Guo & Wang (2008 ▶); Wang, Liu & Cao (2008 ▶).
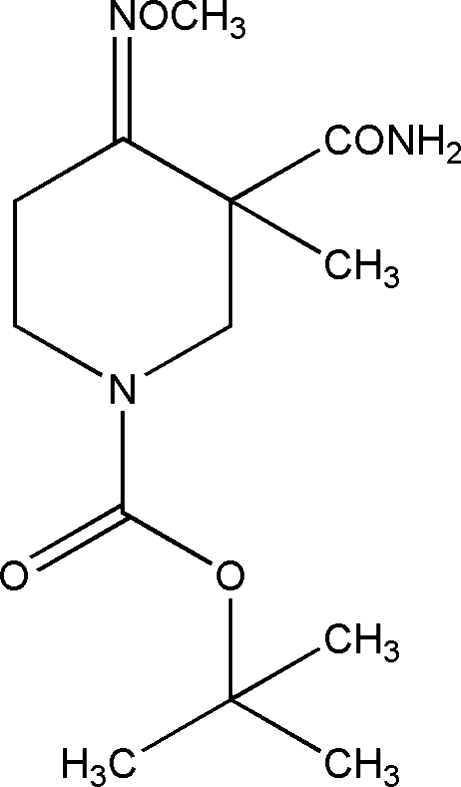


## Experimental

### 

#### Crystal data

C_13_H_23_N_3_O_4_
*M* *_r_* = 285.34
Triclinic, 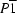

*a* = 7.3750 (14) Å
*b* = 10.0132 (16) Å
*c* = 11.3383 (18) Å
α = 79.5710 (10)°
β = 73.0340 (10)°
γ = 84.973 (2)°
*V* = 787.1 (2) Å^3^
*Z* = 2
Mo *K*α radiation
μ = 0.09 mm^−1^
*T* = 298 (2) K
0.50 × 0.45 × 0.44 mm

#### Data collection

Bruker SMART APEX CCD diffractometer
Absorption correction: multi-scan (**SADABS**; Sheldrick, 1996 ▶) *T*_min_ = 0.946, *T*_max_ = 0.963
4100 measured reflections
2727 independent reflections
1535 reflections with *I* > 2σ(*I*)
*R*_int_ = 0.041

#### Refinement

*R*[*F* ^2^ > 2σ(*F* ^2^)] = 0.056
*wR*(*F* ^2^) = 0.176
*S* = 1.04
2727 reflections
187 parameters
H-atom parameters constrained
Δρ_max_ = 0.22 e Å^−3^
Δρ_min_ = −0.18 e Å^−3^

### 

Data collection: *SMART* (Bruker, 1998 ▶); cell refinement: *SAINT* (Bruker, 1999 ▶); data reduction: *SAINT*; program(s) used to solve structure: *SHELXS97* (Sheldrick, 2008 ▶); program(s) used to refine structure: *SHELXL97* (Sheldrick, 2008 ▶); molecular graphics: *SHELXTL* (Sheldrick, 2008 ▶); software used to prepare material for publication: *SHELXTL*.

## Supplementary Material

Crystal structure: contains datablocks global, I. DOI: 10.1107/S1600536809000634/is2378sup1.cif

Structure factors: contains datablocks I. DOI: 10.1107/S1600536809000634/is2378Isup2.hkl

Additional supplementary materials:  crystallographic information; 3D view; checkCIF report

## Figures and Tables

**Table 1:** Hydrogen-bond geometry (Å, °)

*D*—H⋯*A*	*D*—H	H⋯*A*	*D*⋯*A*	*D*—H⋯*A*
N2—H2*B*⋯O2	0.86	2.25	3.026 (3)	150
N2—H2*A*⋯O3^i^	0.86	2.06	2.913 (3)	173

Symmetry code: (i) 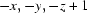.

**Fig. 1.**
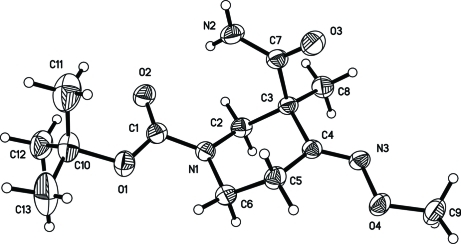
The molecular structure of (I), showing 40% probability displacement ellipsoids and the atom-numbering scheme.

**Fig. 2.**
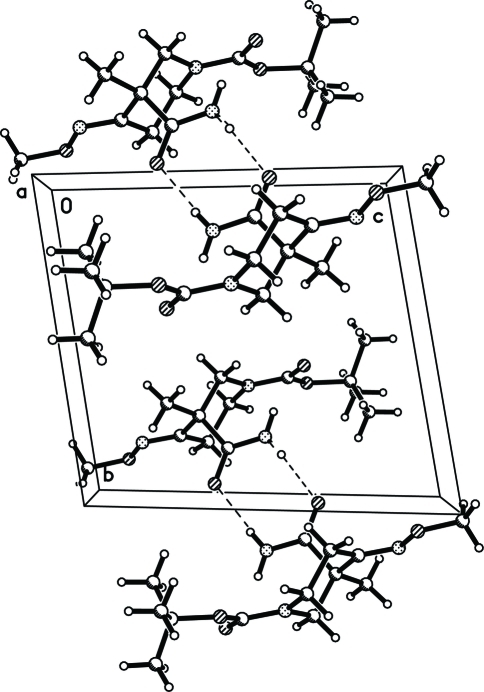
The crystal packing of the title compound.

## supplementary crystallographic information

### Comment

Quinolone antibacterial agents have emerged as one of the dominant classes of chemotherapeutic drugs for the treatment of various bacterial infections in both community and hospital settings (Ray *et al.*, 2005; Ball *et al.*, 1998). In general, 5- and 6 -membered nitrogen heterocycles including piperazinyl, pyrrolidinyl and piperidinyl type side chains have been proven to be the optimal substituents (Anderson & Osheroff, 2001; Choi *et al.*, 2004). Recently, as part of an ongoing program to find potent new quinolones displaying strong Gram-positive activity, we have focused our attention on introducing new functional groups to the piperidine ring (Wang, Guo & Wang, 2008; Wang, Liu & Cao, 2008). We report here the crystal structure of the title compound, which is a key intermediate of 3-amino-4-methoxyimino-3-methylpiperidine, a novel C-7 substituent of the quinolones.

In the molecule of the title compound (Fig. 1), the N1—C1 [1.352 (3) Å] and N2—C7 [1.320 (3) Å] bond lengths are significantly shorter than the normal C—N single bond (1.47 Å), indicating some conjugation with the C1═O2 and C7═O3 carbonyl groups, respectively. The six-membered piperidine ring adopts a chair conformation. In the crystal structure, the molecules have an intramolecular N—H···O and an intermolecular N—H···O hydrogen bond (Table 1 & Fig. 2)

### Experimental

The title compound was prepared from methyl *N*-*tert*-butoxycarbonyl-4- methoxyimino-3-methylpiperidine-3-carboxylate. To a stirring solution of methyl *N*-*tert*-butoxycarbonyl-4-methoxyimino-3-methylpiperidine-3-carboxylate (17.00 g, 56.6 mmol) in methanol (100 ml) was added dropwise a solution of sodium hydroxide (4.53 g, 113.2 mmol) dissolved in distilled water (20 ml) at room temperature. The reaction mixture was heated to 50 °C and stirred for 2 h at the same temperature. After removal of the methanol under reduced pressure, the reaction mixture was diluted with distilled water (30 ml), adjusted to pH 6.0–6.5 with acetic acid. The solid collected by suction was dissolved in methylene chloride (150 ml), and to this solution was added triethylamine (8.8 ml, 63.6 mmol). The reaction mixture was cooled to -14 °C, using an ice-salt bath, isobutyl chloroformate (9.0 ml, 69.2 mmol) was added and stirred for 0.5 h at the same temperature, pumped ammonia gas cautiously at 0–5 °C for 0.5 h, washed with 1 N HCl and saturated brine, respectively, dried over anhydrous sodium sulfate and concentrated under reduced pressure. The resulting yellow residue was recrystallized from ethyl acetate to give the title compound (13.50 g, 83.6%; m.p. 126–127 °C) as a white solid. Single crystals suitable for X-ray analysis were obtained by slow evaporation of an ethyl acetate solution. ^1^H NMR (CDCl_3_, δ): 1.37 (3*H*, s, CH_3_), 1.47 (9*H*, s, CH_3_), 2.14–3.86 (4*H*, m, C_5_, C_6_), 3.89 (3*H*, s, OCH_3_), 4.35–4.37 (2*H*, m,*C*_2_), 5.37 (1*H*, br, CONH), 6.19 (1*H*, br, CONH). MS (ESI, m/*z*): 286 (*M*+H)^+^.

### Refinement

All H atoms were placed at calculated positions, with C—H = 0.96–0.97Å and N—H= 0.86 Å, and were included in the final cycles of refinement using a riding model, with *U*_iso_(H) = 1.2*U*_eq_(C, N) or 1.5*U*_eq_(methyl C), allowing for free rotation of the methyl groups.

### Crystal data

**Table:**

C_13_H_23_N_3_O_4_	*Z* = 2
*M**_r_* = 285.34	*F*(000) = 308
Triclinic, *P*1	*D*_x_ = 1.204 Mg m^−^^3^
Hall symbol: -P 1	Mo *K*α radiation, λ = 0.71073 Å
*a* = 7.3750 (14) Å	Cell parameters from 1147 reflections
*b* = 10.0132 (16) Å	θ = 2.6–23.6°
*c* = 11.3383 (18) Å	µ = 0.09 mm^−^^1^
α = 79.571 (1)°	*T* = 298 K
β = 73.034 (1)°	Block, colorless
γ = 84.973 (2)°	0.50 × 0.45 × 0.44 mm
*V* = 787.1 (2) Å^3^	

### Data collection

**Table:**

Bruker SMART APEX CCD diffractometer	2727 independent reflections
Radiation source: fine-focus sealed tube	1535 reflections with *I* > 2σ(*I*)
graphite	*R*_int_ = 0.041
φ and ω scans	θ_max_ = 25.0°, θ_min_ = 1.9°
Absorption correction: multi-scan (*SADABS*; Sheldrick, 1996)	*h* = −7→8
*T*_min_ = 0.946, *T*_max_ = 0.963	*k* = −11→10
4100 measured reflections	*l* = −12→13

### Refinement

**Table:**

Refinement on *F*^2^	Secondary atom site location: difference Fourier map
Least-squares matrix: full	Hydrogen site location: inferred from neighbouring sites
*R*[*F*^2^ > 2σ(*F*^2^)] = 0.056	H-atom parameters constrained
*wR*(*F*^2^) = 0.176	*w* = 1/[σ^2^(*F*_o_^2^) + (0.0793*P*)^2^] where *P* = (*F*_o_^2^ + 2*F*_c_^2^)/3
*S* = 1.04	(Δ/σ)_max_ = 0.001
2727 reflections	Δρ_max_ = 0.22 e Å^−^^3^
187 parameters	Δρ_min_ = −0.18 e Å^−^^3^
0 restraints	Extinction correction: *SHELXL97* (Sheldrick, 2008), Fc^*^=kFc[1+0.001xFc^2^λ^3^/sin(2θ)]^-1/4^
Primary atom site location: structure-invariant direct methods	Extinction coefficient: 0.052 (9)

### Special details

**Table:**

Geometry. All e.s.d.'s (except the e.s.d. in the dihedral angle between two l.s. planes) are estimated using the full covariance matrix. The cell e.s.d.'s are taken into account individually in the estimation of e.s.d.'s in distances, angles and torsion angles; correlations between e.s.d.'s in cell parameters are only used when they are defined by crystal symmetry. An approximate (isotropic) treatment of cell e.s.d.'s is used for estimating e.s.d.'s involving l.s. planes.
Refinement. Refinement of *F*^2^ against ALL reflections. The weighted *R*-factor *wR* and goodness of fit *S* are based on *F*^2^, conventional *R*-factors *R* are based on *F*, with *F* set to zero for negative *F*^2^. The threshold expression of *F*^2^ > σ(*F*^2^) is used only for calculating *R*-factors(gt) *etc*. and is not relevant to the choice of reflections for refinement. *R*-factors based on *F*^2^ are statistically about twice as large as those based on *F*, and *R*- factors based on ALL data will be even larger.

### Fractional atomic coordinates and isotropic or equivalent isotropic displacement parameters (Å^2^)

**Table:**

	*x*	*y*	*z*	*U*_iso_*/*U*_eq_	
N1	0.3951 (3)	0.3430 (2)	0.4995 (2)	0.0486 (6)	
N2	0.0625 (3)	0.1810 (2)	0.4744 (2)	0.0647 (8)	
H2A	0.0302	0.1251	0.4355	0.078*	
H2B	0.0918	0.2623	0.4374	0.078*	
N3	0.1916 (3)	0.1524 (2)	0.8514 (2)	0.0485 (6)	
O1	0.6155 (3)	0.3495 (2)	0.31633 (18)	0.0636 (6)	
O2	0.3110 (3)	0.4119 (2)	0.32090 (19)	0.0649 (6)	
O3	0.0266 (4)	0.0264 (2)	0.6479 (2)	0.0825 (8)	
O4	0.3333 (3)	0.0907 (2)	0.90663 (17)	0.0580 (6)	
C1	0.4323 (4)	0.3706 (3)	0.3737 (3)	0.0504 (7)	
C2	0.2080 (4)	0.3727 (3)	0.5782 (3)	0.0502 (7)	
H2C	0.1290	0.4149	0.5257	0.060*	
H2D	0.2180	0.4373	0.6301	0.060*	
C3	0.1121 (4)	0.2457 (3)	0.6625 (2)	0.0453 (7)	
C4	0.2516 (4)	0.1757 (3)	0.7326 (2)	0.0426 (7)	
C5	0.4480 (4)	0.1488 (3)	0.6534 (3)	0.0533 (8)	
H5A	0.4454	0.0802	0.6036	0.064*	
H5B	0.5289	0.1142	0.7067	0.064*	
C6	0.5292 (4)	0.2785 (3)	0.5672 (3)	0.0549 (8)	
H6A	0.5545	0.3409	0.6163	0.066*	
H6B	0.6480	0.2567	0.5082	0.066*	
C7	0.0673 (4)	0.1420 (3)	0.5911 (3)	0.0523 (7)	
C8	−0.0772 (4)	0.2898 (3)	0.7492 (3)	0.0632 (9)	
H8A	−0.1396	0.2111	0.8017	0.095*	
H8B	−0.1565	0.3366	0.6999	0.095*	
H8C	−0.0538	0.3494	0.8003	0.095*	
C9	0.2502 (5)	0.0608 (4)	1.0379 (3)	0.0750 (10)	
H9A	0.2237	0.1438	1.0719	0.112*	
H9B	0.3364	0.0036	1.0756	0.112*	
H9C	0.1342	0.0148	1.0548	0.112*	
C10	0.6835 (5)	0.3554 (3)	0.1795 (3)	0.0698 (10)	
C11	0.5789 (7)	0.2537 (4)	0.1411 (4)	0.1179 (17)	
H11A	0.5946	0.1648	0.1865	0.177*	
H11B	0.6297	0.2525	0.0530	0.177*	
H11C	0.4464	0.2796	0.1594	0.177*	
C12	0.6609 (5)	0.4976 (3)	0.1139 (3)	0.0817 (11)	
H12A	0.5286	0.5235	0.1307	0.123*	
H12B	0.7158	0.5015	0.0255	0.123*	
H12C	0.7241	0.5588	0.1436	0.123*	
C13	0.8911 (6)	0.3152 (5)	0.1616 (4)	0.1190 (17)	
H13A	0.9503	0.3801	0.1898	0.179*	
H13B	0.9521	0.3132	0.0746	0.179*	
H13C	0.9025	0.2267	0.2090	0.179*	

### Atomic displacement parameters (Å^2^)

**Table:**

	*U*^11^	*U*^22^	*U*^33^	*U*^12^	*U*^13^	*U*^23^
N1	0.0479 (14)	0.0503 (14)	0.0474 (14)	−0.0055 (11)	−0.0176 (11)	0.0010 (11)
N2	0.088 (2)	0.0558 (15)	0.0618 (17)	−0.0246 (13)	−0.0409 (15)	0.0035 (12)
N3	0.0467 (14)	0.0515 (14)	0.0507 (15)	0.0004 (10)	−0.0223 (12)	−0.0041 (11)
O1	0.0670 (15)	0.0675 (14)	0.0497 (13)	−0.0009 (11)	−0.0118 (11)	−0.0012 (10)
O2	0.0748 (15)	0.0634 (13)	0.0592 (13)	−0.0091 (11)	−0.0332 (12)	0.0098 (10)
O3	0.130 (2)	0.0610 (15)	0.0685 (15)	−0.0418 (14)	−0.0493 (15)	0.0113 (11)
O4	0.0497 (12)	0.0750 (14)	0.0472 (12)	0.0076 (10)	−0.0202 (9)	0.0013 (10)
C1	0.060 (2)	0.0407 (16)	0.0516 (18)	−0.0073 (14)	−0.0207 (16)	0.0003 (13)
C2	0.0539 (18)	0.0430 (16)	0.0570 (18)	0.0003 (13)	−0.0233 (15)	−0.0048 (13)
C3	0.0460 (16)	0.0454 (15)	0.0483 (16)	−0.0040 (12)	−0.0214 (13)	−0.0028 (12)
C4	0.0469 (16)	0.0385 (14)	0.0454 (17)	−0.0041 (12)	−0.0180 (13)	−0.0050 (12)
C5	0.0521 (17)	0.0562 (18)	0.0506 (17)	0.0067 (14)	−0.0195 (14)	−0.0023 (14)
C6	0.0487 (17)	0.0651 (19)	0.0513 (18)	−0.0047 (14)	−0.0197 (14)	−0.0001 (14)
C7	0.0566 (18)	0.0502 (18)	0.0550 (19)	−0.0094 (14)	−0.0271 (15)	0.0013 (14)
C8	0.0476 (18)	0.072 (2)	0.066 (2)	0.0032 (15)	−0.0190 (15)	−0.0008 (16)
C9	0.067 (2)	0.106 (3)	0.0469 (19)	0.0100 (19)	−0.0218 (17)	0.0019 (18)
C10	0.091 (3)	0.061 (2)	0.0489 (19)	−0.0032 (18)	−0.0078 (18)	−0.0056 (15)
C11	0.183 (5)	0.084 (3)	0.081 (3)	−0.036 (3)	−0.008 (3)	−0.031 (2)
C12	0.109 (3)	0.071 (2)	0.059 (2)	−0.018 (2)	−0.020 (2)	0.0060 (17)
C13	0.123 (4)	0.124 (4)	0.072 (3)	0.029 (3)	0.012 (3)	0.001 (2)

### Geometric parameters (Å, °)

**Table:**

N1—C1	1.352 (3)	C5—H5B	0.9700
N1—C2	1.447 (3)	C6—H6A	0.9700
N1—C6	1.461 (3)	C6—H6B	0.9700
N2—C7	1.320 (3)	C8—H8A	0.9600
N2—H2A	0.8600	C8—H8B	0.9600
N2—H2B	0.8600	C8—H8C	0.9600
N3—C4	1.274 (3)	C9—H9A	0.9600
N3—O4	1.412 (3)	C9—H9B	0.9600
O1—C1	1.334 (3)	C9—H9C	0.9600
O1—C10	1.477 (4)	C10—C12	1.502 (4)
O2—C1	1.220 (3)	C10—C13	1.512 (5)
O3—C7	1.234 (3)	C10—C11	1.522 (5)
O4—C9	1.421 (3)	C11—H11A	0.9600
C2—C3	1.534 (3)	C11—H11B	0.9600
C2—H2C	0.9700	C11—H11C	0.9600
C2—H2D	0.9700	C12—H12A	0.9600
C3—C4	1.526 (3)	C12—H12B	0.9600
C3—C8	1.536 (4)	C12—H12C	0.9600
C3—C7	1.537 (4)	C13—H13A	0.9600
C4—C5	1.496 (4)	C13—H13B	0.9600
C5—C6	1.528 (4)	C13—H13C	0.9600
C5—H5A	0.9700		
			
C1—N1—C2	120.1 (2)	O3—C7—N2	122.2 (3)
C1—N1—C6	125.2 (2)	O3—C7—C3	118.3 (2)
C2—N1—C6	114.7 (2)	N2—C7—C3	119.4 (2)
C7—N2—H2A	120.0	C3—C8—H8A	109.5
C7—N2—H2B	120.0	C3—C8—H8B	109.5
H2A—N2—H2B	120.0	H8A—C8—H8B	109.5
C4—N3—O4	112.3 (2)	C3—C8—H8C	109.5
C1—O1—C10	121.1 (2)	H8A—C8—H8C	109.5
N3—O4—C9	108.1 (2)	H8B—C8—H8C	109.5
O2—C1—O1	125.0 (3)	O4—C9—H9A	109.5
O2—C1—N1	123.0 (3)	O4—C9—H9B	109.5
O1—C1—N1	112.0 (3)	H9A—C9—H9B	109.5
N1—C2—C3	112.9 (2)	O4—C9—H9C	109.5
N1—C2—H2C	109.0	H9A—C9—H9C	109.5
C3—C2—H2C	109.0	H9B—C9—H9C	109.5
N1—C2—H2D	109.0	O1—C10—C12	110.4 (3)
C3—C2—H2D	109.0	O1—C10—C13	101.3 (3)
H2C—C2—H2D	107.8	C12—C10—C13	110.6 (3)
C4—C3—C2	106.7 (2)	O1—C10—C11	109.4 (3)
C4—C3—C8	113.3 (2)	C12—C10—C11	112.3 (3)
C2—C3—C8	108.6 (2)	C13—C10—C11	112.2 (3)
C4—C3—C7	107.2 (2)	C10—C11—H11A	109.5
C2—C3—C7	114.1 (2)	C10—C11—H11B	109.5
C8—C3—C7	107.1 (2)	H11A—C11—H11B	109.5
N3—C4—C5	127.1 (2)	C10—C11—H11C	109.5
N3—C4—C3	117.0 (2)	H11A—C11—H11C	109.5
C5—C4—C3	115.8 (2)	H11B—C11—H11C	109.5
C4—C5—C6	110.8 (2)	C10—C12—H12A	109.5
C4—C5—H5A	109.5	C10—C12—H12B	109.5
C6—C5—H5A	109.5	H12A—C12—H12B	109.5
C4—C5—H5B	109.5	C10—C12—H12C	109.5
C6—C5—H5B	109.5	H12A—C12—H12C	109.5
H5A—C5—H5B	108.1	H12B—C12—H12C	109.5
N1—C6—C5	110.2 (2)	C10—C13—H13A	109.5
N1—C6—H6A	109.6	C10—C13—H13B	109.5
C5—C6—H6A	109.6	H13A—C13—H13B	109.5
N1—C6—H6B	109.6	C10—C13—H13C	109.5
C5—C6—H6B	109.6	H13A—C13—H13C	109.5
H6A—C6—H6B	108.1	H13B—C13—H13C	109.5
			
C4—N3—O4—C9	−176.3 (2)	C2—C3—C4—C5	−51.9 (3)
C10—O1—C1—O2	−9.5 (4)	C8—C3—C4—C5	−171.3 (2)
C10—O1—C1—N1	171.7 (2)	C7—C3—C4—C5	70.7 (3)
C2—N1—C1—O2	−5.1 (4)	N3—C4—C5—C6	−124.0 (3)
C6—N1—C1—O2	172.4 (3)	C3—C4—C5—C6	52.5 (3)
C2—N1—C1—O1	173.8 (2)	C1—N1—C6—C5	−122.0 (3)
C6—N1—C1—O1	−8.8 (4)	C2—N1—C6—C5	55.6 (3)
C1—N1—C2—C3	119.1 (3)	C4—C5—C6—N1	−50.8 (3)
C6—N1—C2—C3	−58.6 (3)	C4—C3—C7—O3	47.7 (3)
N1—C2—C3—C4	52.9 (3)	C2—C3—C7—O3	165.5 (3)
N1—C2—C3—C8	175.4 (2)	C8—C3—C7—O3	−74.2 (3)
N1—C2—C3—C7	−65.3 (3)	C4—C3—C7—N2	−136.3 (3)
O4—N3—C4—C5	−2.0 (4)	C2—C3—C7—N2	−18.5 (4)
O4—N3—C4—C3	−178.5 (2)	C8—C3—C7—N2	101.7 (3)
C2—C3—C4—N3	124.9 (2)	C1—O1—C10—C12	66.9 (3)
C8—C3—C4—N3	5.5 (3)	C1—O1—C10—C13	−175.9 (3)
C7—C3—C4—N3	−112.4 (3)	C1—O1—C10—C11	−57.2 (4)

### Hydrogen-bond geometry (Å, °)

**Table:**

*D*—H···*A*	*D*—H	H···*A*	*D*···*A*	*D*—H···*A*
N2—H2B···O2	0.86	2.25	3.026 (3)	150
N2—H2A···O3^i^	0.86	2.06	2.913 (3)	173

Symmetry codes: (i) −*x*, −*y*, −*z*+1.
